# Virtual clinical pharmacy services: A model of care to improve medication safety in rural and remote Australian health services

**DOI:** 10.1093/ajhp/zxac082

**Published:** 2022-03-15

**Authors:** Brett Chambers, Cristen Fleming, Anna Packer, Louis Botha, Gerard Hawthorn, Shannon Nott

**Affiliations:** Western NSW Local Health District, Dubbo, Australia; Western NSW Local Health District, Dubbo, Australia; Western NSW Local Health District, Dubbo, Australia; Western NSW Local Health District, Orange, Australia; Western NSW Local Health District, Orange, Australia; Western NSW Local Health District, Dubbo, Australia

**Keywords:** hospital pharmacy service, rural health, rural hospital, telemedicine, telepharmacy, virtual pharmacy

## Abstract

**Purpose:**

To describe a virtual clinical pharmacy service as a model of care to support rural and remote Australian hospitals that otherwise would not have access to onsite pharmacists.

**Summary:**

Many small hospitals in Australia do not have an onsite hospital pharmacist and struggle to support and optimize patient care. To increase access to a hospital pharmacist’s specialized skills and medication knowledge, a virtual clinical pharmacy service was designed and implemented in 8 hospitals across rural New South Wales, Australia in 2020. The virtual clinical pharmacy service focuses on the core role of hospital pharmacists, including obtaining a best possible medication history, medication reconciliation at transitions of care, medication review, interprofessional team meetings, provision of patient-friendly medication lists, antimicrobial stewardship, and patient and clinician education. The model is aligned with recognized standards of practice for the delivery of clinical pharmacy services in Australian hospitals. This article details a model of care for translation across other settings. It provides the necessary details on clinical services, processes, supporting structures, an evaluation framework, and other important considerations for implementing virtual pharmacy services.

**Conclusion:**

This research provides policymakers, health service planners, and practitioners with a model for providing comprehensive clinical pharmacy services virtually to increase the safe and effective use of medicines. Future publication of the findings of a formal evaluation of the model’s acceptability and effectiveness is planned.

**Key Points**

- Virtual clinical pharmacy involves the use of telehealth technologies to improve access to the skills and knowledge of clinical pharmacists in rural and remote hospitals.
- Virtual pharmacy integrates pharmacists into healthcare teams and complements existing medication management activities.
- Virtual pharmacy supports the safe and effective use of medications and may improve compliance with accreditation standards in smaller resource-poor hospitals.

In New South Wales (NSW), Australia’s most urbanized state, Western NSW Local Health District (WNSWLHD) and Far West Local Health District (FWLHD) are responsible for managing public hospitals and health services within the state’s most rural and remote communities. Together, they service 4% of the state’s population (309,100 people) across 55% of the state’s geographic area (433,379 square kilometers) which is roughly the size of California. Rural Australians living in these Local Health Districts, like their peers nationally and worldwide, experience poorer health outcomes and higher rates of avoidable hospitalization, death, and injury than those living in metropolitan areas.^1^ Despite these significant health needs, the number of health professionals decreases with remoteness, resulting in reduced availability of clinical services and specialized treatments.^1^

Medication-related harm represents a substantial global burden on patients and healthcare systems that is potentially avoidable.^2^ In a hospital setting, clinical pharmacists aim to reduce medication misadventure through interventions such as medication reconciliation, focused education, and minimizing polypharmacy.^3-5^ However, small-community rural hospitals commonly do not have access to clinical pharmacists, particularly in facilities that have low inpatient numbers (<5 patients), have low numbers of emergency presentations (<10 patients per day) and are geographically isolated. This is the case in WNSWLHD and FWLHD, with only 8 of the region’s 47 hospitals having onsite clinical pharmacy support.

A virtual clinical pharmacy service (VCPS) was established as part of a research project to address those challenges in 8 small rural and remote hospitals. Criteria for inclusion included rural and remote hospital location, higher patient activity, electronic medication management (eMeds) and no routine clinical pharmacy support.^6^ Two hospitals were located in FWLHD and 6 hospitals in WNSWLHD, with the local government area population ranging from 2,268 people in the smallest community to 6,794 people in the largest community.^7^ The VCPS was established through a state government translational research grant to improve medication safety and evaluate the acceptability and effectiveness (Table 1) of clinical pharmacy services delivered via telehealth, with data collected between April 2020 and June 2021 (Table 2).^6^ While the activities of the VCPS align with face-to-face pharmacy services, there are essential enablers and challenges to be considered during design and implementation (Figure 1). This article describes the model of care (MoC), including the setting, process of care, supporting technologies, evaluation framework, and shared learnings, for implementing virtual clinical pharmacy into rural hospital practice.

**Table 1.** Program Logic Summarizing Activities, Mechanism of Change, and Monitoring and Evaluation Measures for a Virtual Clinical Pharmacy Service

Multicomponent Virtual Pharmacy Intervention		Mechanisms of Change	Measures
Core Components	Description		
Medication reconciliation	• Pharmacist conducts medication reconciliation on admission, discharge, or transfer • Improve continuity of care through liaising with community health providers	• Increased medication reconciliationcompletion • Improved discharge processes from facility back to community health providers	• No. of medication management plans • Rates of medication reconciliation on admission • Rates of medication reconciliation on discharge
Medication review	• Pharmacist review of patients on high-risk medications • Identify drug-related causes of admission • Therapeutic drug monitoring of narrow therapeutic index drugs • Optimizing VTE prophylaxis • Detection of medication interactions • Optimizing chronic disease management • Providing antimicrobial stewardship review • Review of patients’ falls risk	• Increased clinical activities will optimize patient medication management during admission to a health facility	• No. of medication reviews • No. of AMS reviews • Antimicrobial usage rates • VTE prophylaxis rates • No. of clinical interventions and/or recommendations
Patient education	• Counseling on new medications and medication changes • Provision of patient-friendly medication list • Provision of consumer medication information	• Enhanced patient understanding about medication and potential side effects via counseling and patient friendly medication lists	• No. of medication lists on discharge • No. of medication counseling sessions • Time taken to undertake patient education • Patient-reported experience measures survey
Health facility engagement	• Site visits • Service rounding • Staff education • Newsletters • Posters	• Building trust and engagement will ensure staff view the VCPS as a resource to help them achieve safe and effective use of medicines	• No. of site referrals • No. of video consultations • Staff attendance at medication education sessions • Uptake of pharmacy recommendations

Abbreviations: VCPS, virtual clinical pharmacy service; VTE, venous thromboembolism.

**Table 2.** Activity Measures for Virtual Clinical Pharmacy Service at 8 Rural Australian Hospitals Over 12-Month Period

Measure	Count (*n* = 1,306 Patients)
Site-requested consults completed	574
Medication management plans	1,406
Clinical and medication review	4,406
Discharge medication review	580
Patient medication list	629
Clinical interventions	2,514
Total pharmacy occasions of care	7,021

**Figure 1.**
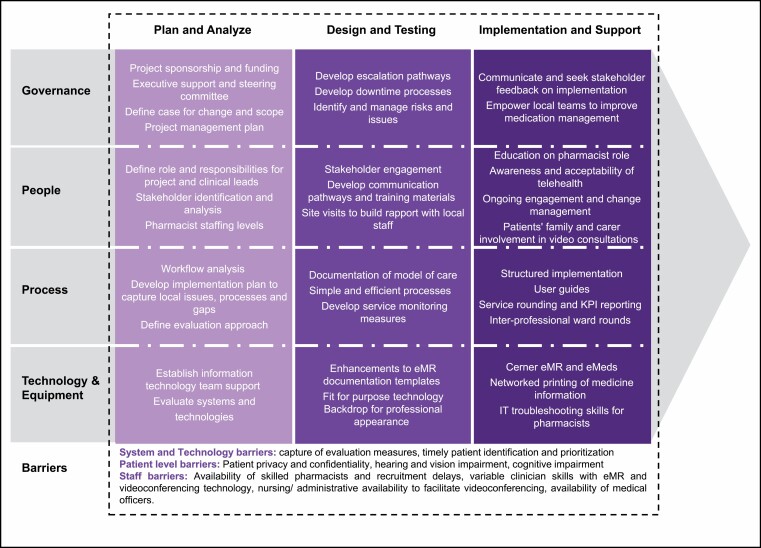
Simplified project plan with key considerations and barriers to implementing a virtual clinical pharmacy service. eMeds indicates electronic mediation management; EMR, electronic medical record; VC, videoconferencing.

## The VCPS model

An interdisciplinary team including senior health administrators, physicians, nurses, pharmacists, and researchers designed the VCPS MoC to provide high-quality, accessible, and sustainable virtual pharmacy services. The model integrates virtual pharmacists into the healthcare team to complement and improve existing medication management and assist hospitals in meeting national medication safety standards.^8^

Australian standards of practice for delivering clinical pharmacy services provide the basis for the activities provided under this model.^9^ In the project described here, the VCPS employed 2.1 Australia-registered clinical pharmacist full-time equivalents to serve an average of 47 occupied hospital beds, with surge capacity to cover up to 66 beds. In the 2020 and 2021 financial years, activity at each hospital ranged from 2 to 7 admitted patients and 3 to 10 emergency presentations per day. Distance to the nearest hospital with onsite clinical pharmacy services ranged from 30 km to 370 km. The VCPS pharmacists provided medication management services from 8:00 am to 4:30 pm Monday to Friday, with no after-hours or public holiday support.

**Figure 2.**
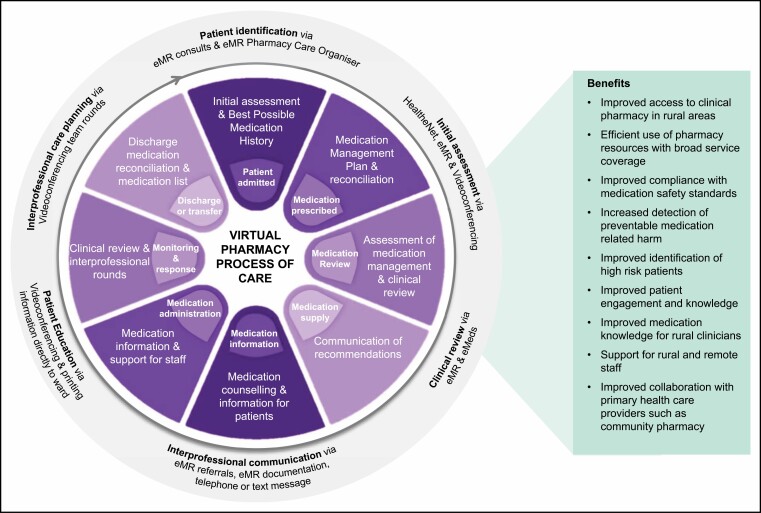
Process of care for delivering a virtual clinical pharmacy service, including facilitating technologies and health-system benefits. eMeds indicates electronic mediation management; EMR, electronic medical record.

The pharmacy team was virtual and decentralized, and staff could reside in the larger regional cities of Dubbo and Orange within WNSWLHD, or outside of these areas on approval. The low patient numbers and vast distance between hospitals made it impractical for pharmacists to travel to each hospital in person. For example, the hospital farthest from a regional referral hospital (370 km away) was 4 hours’ drive via road, and it would take the pharmacist over 23 hours by road, or a 2,136-km round trip, to visit all sites from Dubbo. Utilizing a virtual care model allowed much more efficient use of clinical pharmacist time and increased the reach of clinical pharmacy services across rural and remote hospitals. Use of videoconferencing supported the delivery of core clinical pharmacy services, including collection of a best possible medication history (BPMH), medication reconciliation at transitions of care, medication review, interprofessional team meetings, patient-friendly medication lists, antimicrobial stewardship, and patient and clinician education (Figure 2). The VCPS also utilized advanced practice guidelines for pharmacist-led prescribing of nicotine replacement therapy and provided targeted monthly medication education in response to medication incidents. This model did not include changes in medication supplies, which were maintained through existing processes.

A key component of the VCPS model was the proactive review of patients’ medication therapy. Pharmacists were typically engaged in patient care tasks; however, other quality-focused activities were addressed during periods of lower patient activity, including implementation planning, staff rounding, staff education presentations, and creation of newsletters and monthly performance reports.

### Process of care and facilitating technology

. A vital element of this model was the utilization of digital technologies to deliver an entirely virtual service. To enable this, all health facilities in the region have the Cerner electronic medical record (EMR) (Cerner Corporation, Kansas City, MO), which includes eMeds capabilities. Additionally, purpose-built secure bedside videoconferencing carts were available in all facilities. The pharmacists utilized a clinical workstation comprising a mobile telephone, laptop computer with 2 screens, high-definition webcam and noise-cancelling headset to facilitate reviews via videoconference. Skype for Business (Microsoft Corporation, Redmond, WA) instant messaging was used as the primary communication method within the VCPS team during work shifts. Videoconferencing was chosen to enhance communication with patients and provide a personalized experience. The process of care, facilitating technology, and benefits are summarized in Figure 2 and described below.

#### Step 1: referral and prioritization.

Patient referrals are made by bedside clinicians using a consult request tool in the EMR, and urgent referrals are followed up via a telephone call to a single point-of-contact number. The EMR consult identifies patients for assessment, includes the reason for the request (such as medication reconciliation, medication education, discharge review, or patient medication list) and patient-specific medication risk factors (such as age >65 years, use of >5 medications or >12 medication doses per day, hospital admission in the last 28 days, and taking a high-risk medication). Pharmacists also proactively identify patients using a functional patient list in the EMR called the Pharmacy Care Organiser. The Pharmacy Care Organiser has summary patient information such as length of stay, medication history status, medication reconciliation status, outstanding interventions, and use of high-risk medications. These factors and the stage of a patient’s healthcare journey (ie, admission, transfer, or discharge) enable prioritization of care.

#### Step 2: initial assessment and consultation.

The EMR and My Health Record documentation are utilized to facilitate an initial assessment of a patient’s medications. My Health Record is a national, secure online summary of an individual’s health information across all healthcare providers. It allows healthcare providers to upload health information into one electronic health summary including prescription and dispensing information from both hospital-based and community-based providers. Following review of this information, the pharmacist then contacts the hospital via telephone to coordinate telehealth consultations around other patient care priorities. The Pexip videoconferencing platform (Pexip AS, Oslo, Norway) connects the pharmacist to a mobile videoconferencing device with 2-way audio and video and an integrated tilt, pan, and zoom lens at the patients’ bedside. A telephone consultation is performed in rare instances when telehealth equipment is unavailable or to facilitate infection control measures. A physical branded backdrop behind the pharmacist removes background distractions and provides a professional appearance.

#### Step 3: medication reconciliation and review.

In most circumstances, the initial video consultation includes taking a BPMH, which includes information provided by the patient’s family or caregiver when possible. The pharmacist then communicates any medication-related issues to medical officers or nursing staff via telephone or an electronic consult request and enters comprehensive documentation into the patient’s EMR. Regular medication reviews occur throughout the patient’s admission, and pharmacists follow up medication recommendations on subsequent reviews. Patients can be re-referred to the service throughout their admission to address medication issues, provide education, and assist with discharge planning.

#### Step 4: interprofessional care planning.

During the project, weekly virtual interprofessional ward rounds supported collaborative patient-centered care. These rounds improved care planning by the patient and their family, doctor, nurse(s), and pharmacist, as well as occupational therapy, physiotherapy, speech pathology, dietetics, and social work personnel. Before the VCPS, few hospitals were conducting regular interprofessional ward rounds. The VCPS enhanced existing face-to-face rounds by enabling virtual participation by members of other allied health professions who are not always onsite. Integrated virtual and face-to-face interprofessional rounds are now conducted at most hospitals using the service.

#### Step 5: continuity of medication management.

On discharge, the pharmacist videoconferenced with patients and their families to provide comprehensive medication education. Pharmacists enhanced patient understanding of their medications and changes to their regimen by providing written, patient-friendly medication lists and consumer medicines information. The pharmacist printed out education material directly to the ward via networked printers and liaised with community-based pharmacists to coordinate medication regimen changes.

### Documenting clinical activities.

**Figure 3.**
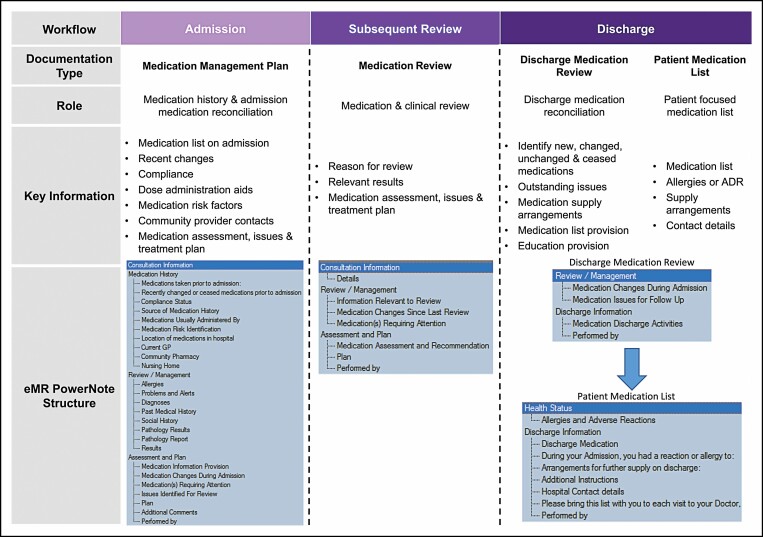
Electronic medical record (EMR) documentation workflow and templates used to document virtual clinical pharmacist consultations. ADR indicates adverse drug reaction.

The VCPS comprehensively documented all patient pharmaceutical care to the EMR and eMeds system. A standardized approach, utilizing documentation templates, ensured other clinicians were able to readily access relevant and up-to-date medication management information. These templates included a medication management plan, medication review, discharge medication review, and patient discharge medication list, which allowed the pharmacist to import relevant information and speed up the documentation process (Figure 3). Comprehensive documentation reduced the need for clinical handoffs within the VCPS team, improved communication, and ensured transparency for all clinicians involved in patient care.

### Evaluation and continuous service improvement.

The VCPS developed and aligned performance indicators with recognized pharmacy standards, healthcare standards, and the organization’s strategic plan. Indicators are used to monitor service utilization, inform quality improvement, and formally evaluate the effectiveness, acceptability, and scalability of virtual pharmacy services in rural and remote hospitals (Table 1).^6^ Managers and researchers used the EMR to collect performance indicators and activity data to minimize the impact on service providers. Between April 27, 2020, and June 30, 2021, the VCPS provided 7,021 occasions of care for 1,306 patients, yielding 2,514 clinical pharmacy interventions (Table 2). An intervention was defined as the process of a pharmacist identifying and making a recommendation to prevent or resolve a drug-related problem.^9^

Doctors, nurses, and allied health professionals endorsed the VCPS, identified benefits, and suggested enhancements in focus groups.^10^ Staff valued having an additional trusted workforce, access to specialized medication advice, improved patient safety, identification of medication errors, and enhanced compliance with antimicrobial stewardship and hospital accreditation standards.^10^ Suggested enhancements included extending service hours and widening patient eligibility.^10^ A formal analysis of this data is underway, with further results to be published separately.

### Benefits.

Telehealth can overcome the challenges of immense distances in Australia, and the benefits are well documented in the literature.^11^ The key benefits realized through the VCPS are equitable access to a pharmacist’s specialized skills and medication knowledge, improved medication safety, improved care coordination, particularly at transitions of care, and compliance with national standards (Figure 2). The service enhanced patient-centered care through structured interprofessional ward rounds and active involvement in medication decision making, and patient feedback suggested high acceptability. Clinicians have reported increased confidence in medication management and safety due to the support provided by pharmacists, and the service has demonstrated scalability over multiple rural and remote facilities.^10^

Since the VCPS implementation, patients in rural and remote communities have received virtual pharmacy services that would otherwise not be available. The service is accessible and responsive to patient care needs and can respond to most requests within the same day. Most hospitals heavily utilize the service, with a very high proportion of patients being served by the VCPS. Rates of BPMH taking, admission and discharge medication reconciliation, and provision of patient-friendly medication lists have all improved.

## Discussion

### Planning, implementation and enablers.

Collaborative development of the MoC and a structured implementation underpin the success of the VCPS. Clinicians, health information communication and technology (HICT) personnel, and health researchers with diverse skills and clinical experience collaborated to design the model and evaluation (Table 1). Representatives from peak health organizations such as the NSW Agency for Clinical Innovation were also engaged and provided strategic advice. Clinical oversight ensured the model drew upon best practice guidelines for clinical service provision and informed pivotal decisions such as determination of staffing ratios. These partnerships ensured processes were streamlined with existing services to simplify workflows for both care providers and receivers. It also provided an opportunity to evaluate technology and systems with subject matter experts and request enhancements to create efficiencies or improve workflow.

An 8-week site implementation plan was an essential tool for planning, engagement, and change management; key aspects of the project planning are summarized in Figure 1. This implementation plan recognized the importance of engaging local stakeholders such as hospital managers, physicians, nurses, and community pharmacists in ensuring successful uptake of the VCPS. These local staff facilitate patient referrals, set up videoconferencing equipment, and support patients and caregivers at the bedside. Also integral to the success of the VCPS was establishing a designated project manager role alongside service-specific pharmacist-lead roles. These positions drove project management, including the design of workflows, embedding change at local facilities and engaging both internal and external stakeholders. Part of the implementation planning included a site visit to provide education on the pharmacist’s role and clinical referral processes, gather information to help understand the local context, promote VCPS benefits, and build rapport with local staff. Reliable technology is critical for a virtual service, and minimal service disruptions were achieved by using fit-for-purpose infrastructure and leveraging a responsive HICT support team. Minimal additional investment in technology was required due to the existing broadband network, extensive telehealth infrastructure, an EMR platform with integrated eMeds capabilities, and access to HICT support.

### Impact of COVID-19.

The research project was envisioned and planned prior to the coronavirus disease 2019 (COVID-19) pandemic; however, the first wave of the pandemic arrived approximately 1 month prior to scheduling of the first hospital VCPS participation. While all sites commenced virtual services as planned, travel restrictions prevented face-to-face site visits for onboarding, and reduced patient presentations slowed initial uptake of the service. Despite those challenges, the VCPS operated continuously throughout the pandemic using a telehealth system access method that allowed ongoing service provision at a time when outreach and in-person healthcare were significantly disrupted. This ability to be COVID-19 resilient also highlights another benefit of VCPS: The ability to mobilize staff via virtual means to the patient’s bedside can facilitate undisrupted clinical care despite external factors (in this case, the COVID-19 pandemic). It is anticipated that this observation would translate to other occasions where face-to-face care would be disrupted, such as natural weather events (eg, wildfires, flooding) that would impede traveling clinicians in accessing rural hospitals.

### Challenges and shared learnings.

As there are fewer opportunistic interactions and conversations with virtual versus in-person care, communication and establishing rapport between the pharmacists and the broader healthcare team is of significant importance. Processes such as assigning a regular pharmacist to each site facilitates relationships, helps build rapport, and provides continuity for staff and patients. Other enablers include a user guide, participation in interprofessional ward rounds, staff education, and service rounding (Figure 1). A newsletter supports communication with staff, and a monthly activity report fosters local ownership of quality improvement. Service rounding involves use of these activity reports to inform discussion of performance indicators, medication safety, antimicrobial stewardship, and continuous service improvement. Service rounding also provides an opportunity to recognize successes and address areas for improvement.

Some patient-specific factors have proven to be challenging in virtual healthcare, including privacy issues, hearing impairment, and impaired cognition. The need for privacy in discussions involving sensitive matters requires careful consideration, as the teleconferencing unit uses speakers, which are often louder than regular in-person conversations. Hearing impairment can be a significant barrier to providing clinical pharmacy when there is no alternative but virtual care. In these circumstances, a family member, caregiver, or onsite clinician to support the patient is required. Generally, videoconference services are not provided to a patient with cognitive impairment unless the primary aim is to discuss care with family, caregivers, or other clinicians. Of note, during the project very few patients declined telehealth pharmacy services.

The limited availability of skilled clinical pharmacists in rural and remote areas is a significant challenge, and there are even fewer pharmacists with experience in the delivery of virtual clinical pharmacy services. This workforce challenge highlights the need for intuitive workflows and comprehensive training for new pharmacists engaged in virtual care. The VCPS model was implemented by an experienced team of senior pharmacists with local knowledge and advanced communication skills, which significantly benefited the service. However, within the established model, relatively junior pharmacists with good communication skills have excelled.

The ad hoc nature of virtual pharmacy can be disruptive for local staff, particularly during times of high activity or low staffing levels. The VCPS is highly reliant on the availability of onsite staff to take telephone or video calls, to arrange patient consultations, and act on clinical recommendations. This challenge is partially mitigated by the pharmacist being aware of local pressures, rearranging consults, and having clear escalation pathways for more urgent issues. Some hospitals opted to utilize administrative staff to facilitate consultations to help reduce nursing workload. This challenge improved organically as nursing and other clinical staff experienced the benefits of virtual pharmacy and the associated time savings.

The ability to timely identify and prioritize patients across multiple hospitals remains a mostly manual and time-consuming process. This suboptimal patient selection remains a challenge despite use of available systems and clinician-led electronic referrals. It is particularly challenging to identify when discharges will occur, which is reflected in VCPS pharmacists’ reviewing significantly fewer patients on discharge (~50%) than at admission (~80%). The identification and prioritization of patients is an element that could be improved in the future to ensure scalability.

### Future of virtual pharmacy.

 Based on the early successes of the VCPS and the transformation of digital healthcare with the COVID-19 pandemic, it is likely that virtual clinical pharmacy will become a more widely accepted MoC. WNSWLHD is in the process of expanding the VCPS to all hospitals in the health district where there is no onsite pharmacy service. This model may be suitable in other contexts, and future research will investigate outcomes of the VCPS model and the feasibility of virtual pharmacy services in an urban environment.

## Conclusion

The VCPS has been established as a scalable MoC to provide clinical pharmacy services to rural and remote health facilities that would otherwise not have access to a pharmacist. It demonstrates a pharmacist can be part of the interprofessional healthcare team along with onsite and other virtual health professionals. This article describes the processes, systems, and enablers that were required for a pharmacist to provide virtual clinical services concurrently to multiple small public hospitals in rural and remote Australia. The VCPS model aims to add to the evidence base for virtual pharmacy service provision to acute hospital inpatients and ensure virtual pharmacy services are appropriately adapted and aligned with recognized standards. A formal evaluation is underway to validate the acceptability, scalability and effectiveness of this model.

## Additional information

Authors’ individual contributions to this work were as follows: Brett Chambers, research coordinator, design of service and evaluation framework, drafting of manuscript, coordination of reviews and publication, and approval of publication; Cristen Fleming, design of service, participation as clinical service lead and provider, critical revision and editing of manuscript, development of graphics, approval of publication; Anna Packer, participation as clinical service provider, critical revision and editing of manuscript, and approval of publication; Louis Botha, participation as clinical service lead and provider, critical revision and editing of manuscript, and approval of publication; Gerard Hawthorn, conception of research, development of grant application, design of service, development of evaluation framework, critical revision and editing of manuscript, and approval of publication; and Shannon Nott, serving as chief investigator, conception of research, development of grant application and evaluation framework, critical revision and editing of manuscript, and approval of publication.
